# Human thirst behavior requires transformation of sensory inputs by intrinsic brain networks

**DOI:** 10.1186/s12915-022-01446-5

**Published:** 2022-11-10

**Authors:** Li-Ming Hsu, Jen-Tsung Yang, Xuyun Wen, Xia Liang, Leng-Chieh Lin, Yen-Chu Huang, Yuan-Hsiung Tsai

**Affiliations:** 1 Center for Animal Magnetic Resonance Imaging, The University of North Carolina at Chapel Hill, Chapel Hill, NC USA; 2 Graduate Institute of Mind, Brain and Consciousness, Taipei Medical University, Taipei, Taiwan; 3 Department of Neurosurgery, Chang Gung Memorial Hospital, Chiayi, Chiayi, Taiwan; 4 College of Computer Science and Technology, Nanjing University of Aeronautics and Astronautics, Nanjing, Jiangsu China; 5 Laboratory for Space Environment and Physical Sciences, Institute of Technology, Harbin, 150001 China; 6 Department of Emergency Medicine, Chang Gung Memorial Hospital, Chiayi, Chiayi, Taiwan; 7 Department of Neurology, Chang Gung Memorial Hospital, Chiayi, Chiayi, Taiwan; 8 Department of Diagnostic Radiology, Chang Gung Memorial Hospital, Chiayi, Chiayi, Taiwan; 9 College of Medicine, Chang Gung University, Taoyuan, Taiwan

**Keywords:** Thirst behavior, Intrinsic network, Functional MRI

## Abstract

**Background:**

To survive and thrive, many animals, including humans, have evolved goal-directed behaviors that can respond to specific physiological needs. An example is thirst, where the physiological need to maintain water balance drives the behavioral basic instinct to drink. Determining the neural basis of such behaviors, including thirst response, can provide insights into the way brain-wide systems transform sensory inputs into behavioral outputs. However, the neural basis underlying this spontaneous behavior remains unclear. Here, we provide a model of the neural basis of human thirst behavior.

**Results:**

We used fMRI, coupled with functional connectivity analysis and serial-multiple mediation analysis, we found that the physiological need for water is first detected by the median preoptic nucleus (MnPO), which then regulates the intention of drinking via serial large-scale spontaneous thought-related intrinsic network interactions that include the default mode network, salience network, and frontal-parietal control network.

**Conclusions:**

Our study demonstrates that the transformation in humans of sensory inputs for a single physiological need, such as to maintain water balance, requires large-scale intrinsic brain networks to transform this input into a spontaneous human behavioral response.

**Supplementary Information:**

The online version contains supplementary material available at 10.1186/s12915-022-01446-5.

## Background

Water is an essential nutrient for every organism because it regulates body temperature, maintains organ functions, and prevents infection. Being sufficiently hydrated is important for improving cognitive functions and maintaining positive moods [1, 2]. To satisfy the water needs of the body, the human brain continuously senses dehydration-related physiological changes to generate a subjective experience of thirst that motivates water intake [3]. Dehydration causes disturbances in the body fluid balance that encompass a spectrum of physiological changes, including increases in blood osmotic pressure and sodium concentration, as well as decreases in blood volume [3–5]. Changes in these physiological parameters are monitored by multiple brain regions, notably the median preoptic nucleus (MnPO) located in the anterior hypothalamus, which integrates osmotic and hormonal signals from the organum vasculosum lamina terminalis (OVLT), subfornical organ (SFO) and peripheral osmoreceptors (including arterial, cardiopulmonary and visceral) and acts as one of the key neural representations of physiological thirst [6, 7].

Subjective thirst that arises from the underlying physiological state has been suggested to occur in a network of higher-order brain regions, including the anterior cingulate cortex (ACC), insula, inferior frontal gyrus, and middle frontal gyrus [8–13]. While the conversion of physiological thirst to subjective thirst is of clear importance [14], it remains unknown how dehydration-related physiological responses integrated in the MnPO are orchestrated with higher-order brain systems to generate subjective experiences of thirst.

Subjective thirst is an internal state that arises from spontaneous thoughts regulated by the salience of specific sensory signals (such as increases in osmolality or decreases in blood volume) [15, 16]. This inference is in concordance with a recently proposed framework for spontaneous thoughts, which suggests that the content of mental states can be dynamically constrained in two ways, either automatic or deliberate [17]. Deliberate constraints are implemented through cognitive control, whereas automatic constraints are caused by affective or sensory salience, uncoupled from the updown cognitive control regulation. Within this framework, subjective thirst may represent a unique case of spontaneous thought that is automatically constrained by physiological thirst signals and deliberately constrained to initiate the motivation to drink.

Building on a growing body of evidence revealing the potential neural basis of spontaneous thought, this dynamic framework also proposed that fluctuations in spontaneous thought and its constraints may be closely linked to the changing interactions among large-scale brain networks [17]. Specifically, the default mode network (DMN), putatively involved in self-referential/internally oriented processes, acts as a source of variability in internally oriented thought over time [18]. The salience network (SN), which is involved in bottom-up salience detection [19], exerts automatic constraints on the output of the DMN, and the frontoparietal control network (FPCN), which implements deliberate constraints by flexibly coupling with the DMN or SN [17], which is closely linked to executive control and goal-directed thought [20, 21]. Therefore, the subjective perception of thirst embedded in the stream of spontaneous thought may manifest as changes in functional communications among these large-scale brain networks, and underlying variations in the interactions between thought content and both automatic and deliberate constraints [22].

**Fig. 1.**
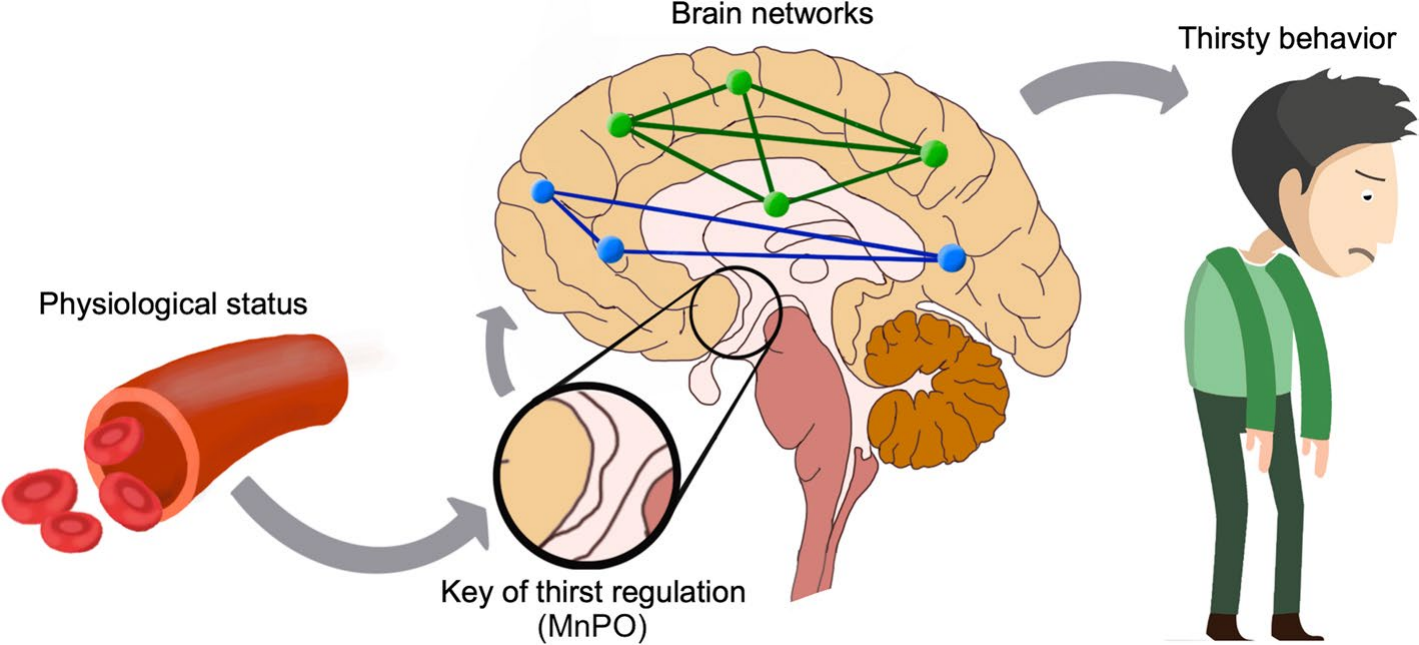
A hypothetical model of the functional neural pathway for the thirst regulation mechanism. In this model, we hypothesized that diverse thirst behaviors are driven by the physiological status through the median preoptic nucleus (MnPO) and three spontaneous thought-related large-scale brain networks, including the default mode network (DMN), salience network (SN), and frontal-parietal control network (FPCN)

**Fig. 2.**
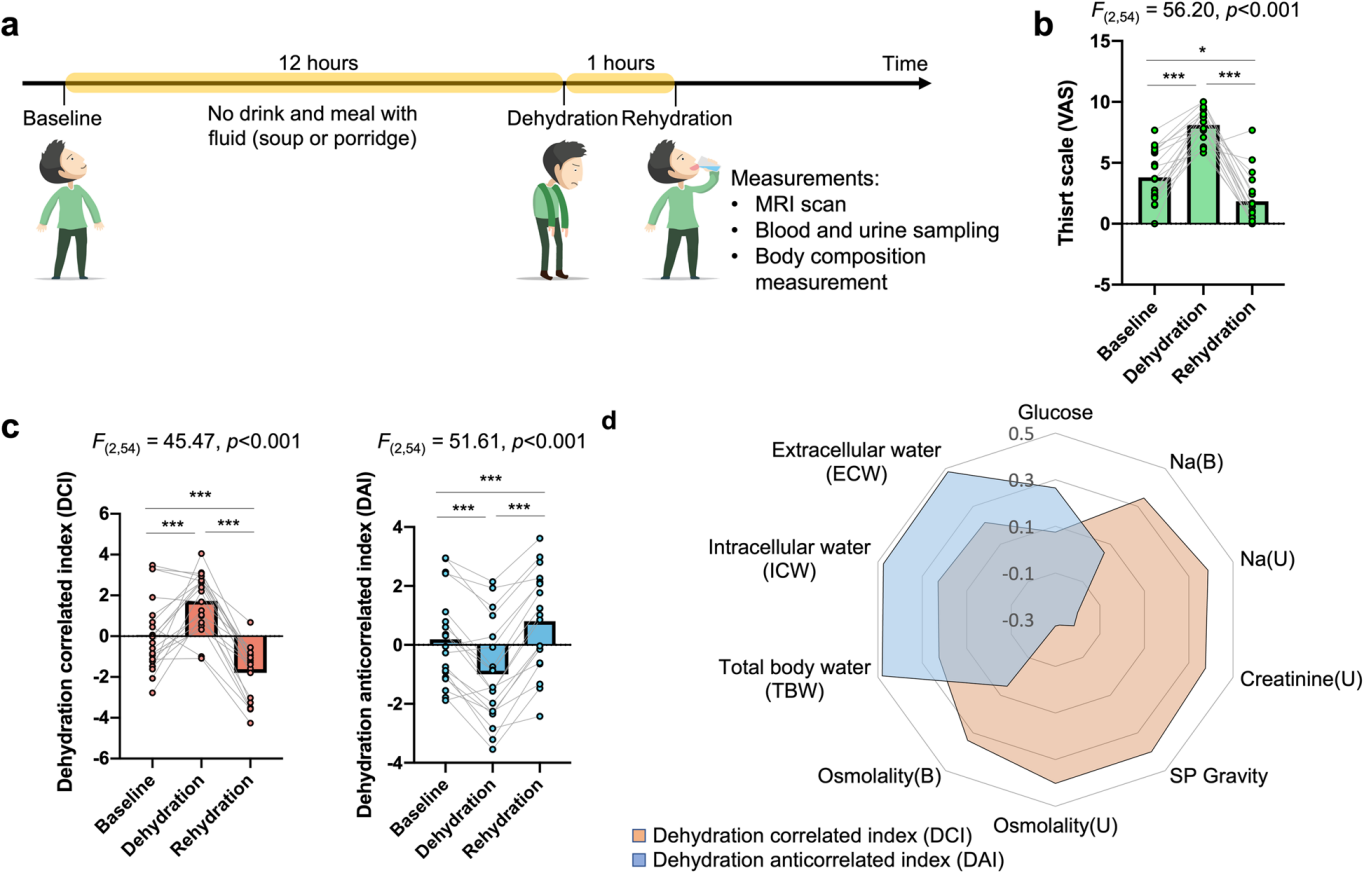
Thirst scale and physiological indices under different hydration statuses. **a** Experimental design of our study. Each subject underwent brain magnetic resonance imaging (MRI) scanning and thirst-related physiological measurements three times within 13 h. The first examination was carried out at the beginning of the experiment, where the participant was in a normohydration state without any limitations on food and fluid intake in the past several hours. After that, the participant was prohibited from eating or drinking anything with water content for 12 h to achieve a dehydration status before participation in the second examination. Subsequently, the participant was asked to drink 1.0 L water for rehydration and completed the final examination within one hour. These examinations include brain MRI scanning and physiological status of hydration evaluation. **b** The VAS showed significant changes among the three statuses in repeated-measures ANOVA. **c** All physiological indices of hydration were normalized to z scores and then pooled into a principal component analysis (PCA) to generate factors that predicted overall hydration physiological status. Two major components with principal eigenvalue scores >1 were extracted and defined: the dehydration-correlated index (DCI) and the dehydration-anticorrelated index (DAI). Both DCI and DAI showed significant changes among the three statuses in repeated-measures ANOVA. **d** The radar plot indicates the principal component coefficients for DCI and DAI in each physiological index. (* *p* value < 0.05, *** *p* value <0.000, FDR corrected)

Taking these considerations together, we propose a model of subjective thirst to offer a synthesized perspective for the understanding of how subjective thirst is intrinsically derived from physiological signals (Fig. 1). In this model, we hypothesized that the subjective experience of thirst might arise from the physiological status through one of the physiological thirst regulating keys (MnPO), which further relays its output to higher-order brain networks that are involved in the coordination of spontaneous thought. To test our hypothesis, we recruited twenty subjects who underwent resting-state fMRI scans during three different hydration statuses (Fig. 2a). Multiple physiological indices of thirst or dehydration, as well as the objective measurement of thirst intensity by a visual analog scale (VAS) thirst scale, were collected in each hydration status for further investigation of the role of neural circuits in processing and transforming the physiological signals into thirst sensation or behavior.

During dehydration and rehydration, we identified significant functional connectivity changes in the MnPO and intrinsic networks compared to normal hydration status (baseline). In addition, to uncover the underlying functional pathways for thirst regulation, we applied serial-multiple mediation analysis to investigate how the association between variations of the subjective thirst scale and the physiological indices was mediated by the changes in interactions between MnPO and these intrinsic networks. Together, our results demonstrate that the altered functional connectivity between the MnPO, SN, DMN, and the FPCN driven by dehydration is a core neural mechanism that underlies the perception of subjective thirst.

## Results

### Alterations of physiological indices and thirst behavior under different hydration statuses

To explore the underlying functional pathway for human thirst regulation, we collected resting-state functional magnetic resonance imaging (rs-fMRI) scans, blood and urine sampling, body composition measurements, and thirst scores for 20 healthy volunteers (10 women and 10 men) with ages ranging from 24 to 40 years (32.1 ± 4.1 years) at baseline (normohydration), dehydration and rehydration (Fig. 2a). One subject was excluded from the following analysis due to the large head motion during MRI scanning (see the “Methods” section). There were significant changes in thirst scale scores among the three hydration statuses (F_(2,54)_ = 56.20, *p*<0.001), with significant differences between baseline and dehydration (*t* = −9.15, *p*<0.001), dehydration and rehydration (*t* = 10.32, *p*<0.001), and baseline and rehydration (*t* = 2.77, *p*<0.05) (Fig. 2b).

To reduce the redundancy of the physiological data (Additional file 1: Fig. S1), we pooled all normalized physiological indices (*z* score) using principal component analysis (PCA) and extracted two major principal components (PCs) with the largest eigenvalues (eigenvalue >1) (Additional file 1: Fig. S2). We projected the physiological indices on these two PCs and explored group differences in PC scores among the three thirst statuses using repeated-measures ANOVA. The first PC scores in dehydration status were significantly higher than those at baseline (*t* = 3.93, *p*<0.001) and in rehydration status (*t* = 13.00, *p*<0.001), while the second PC scores in dehydration status were significantly lower than those at baseline (*t* = −6.16, *p*<0.001) and in rehydration status (*t* = −11.21, *p*<0.001) (Fig. 2c). We thus denoted the first PC as the dehydration-related index (*DCI*) and the second PC as the dehydration-anticorrelated index (*DAI*). In PCA, the *DCI* (44.2%) and *DAI* (32.0%) together accounted for 76.2% of the total variance (Additional file 1: Fig. S2). In addition, based on the magnitude of the principal component coefficients of each input variable in each component, we further found that the *DCI* was mainly contributed to by serum and urine osmolality, serum and urine sodium (Na), urine creatinine and specific gravity (SP gravity), while *DAI* was contributed to by total body water (TBW), intracellular water (ICW), extracellular water (ECW), and serum glucose (Fig. 2d). Notably, the indices that contributed to *DCI* are acquired by the blood test and urinalysis, which estimates the physiological fluid status, while the indices that contributed to DAI are mainly acquired by the bioelectrical impedance analysis, which estimates the body composition. Together, the two major dehydration-related physiological changes (*DCI* and *DAI*) are identified across different dehydration statuses, which represent the physiological fluid statuses and body fluid composition.

### The functional interactions among the MnPO and high-level cognition systems are dynamically regulated in response to continuous thirst-related physiological changes

**Fig. 3.**
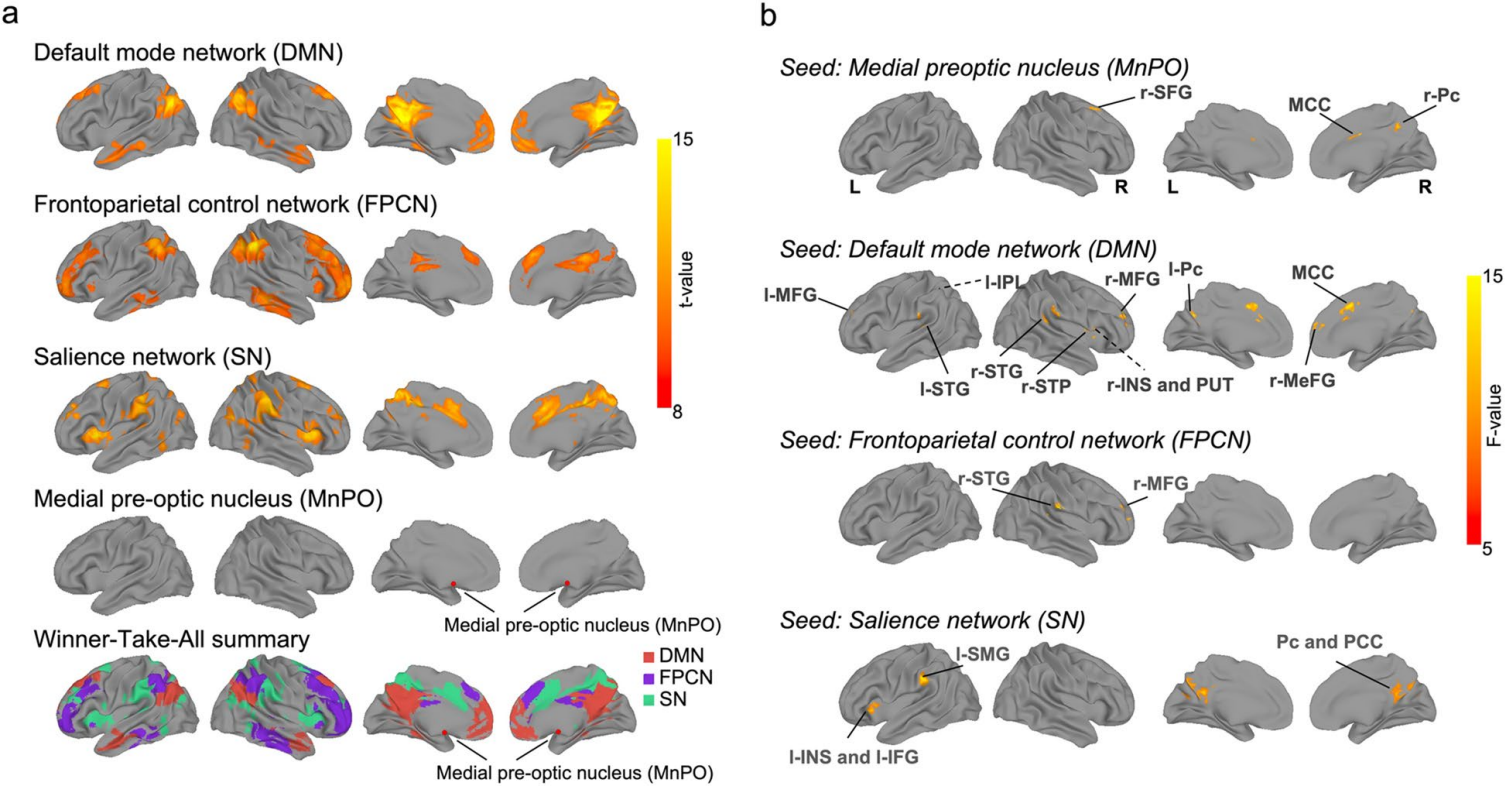
Significant FC changes among identified intrinsic networks and thirst key (median preoptic nucleus, MnPO) across different hydration statuses. **a** MnPo and three intrinsic networks. The seed of the MnPO (MNI coordinate: [3.5, 0.6, −13.2]) was selected manually, and three intrinsic networks (DMN, FPCN, and SN) were identified from 15-component ICA (*t value* > 8, *p*_corrected_ < 0.001, corrected by 3dClustSim in AFNI). A winner-take-all (WTA) summary across all networks was computed and displayed. Each voxel was assigned a color based on the network with the highest t value. **b** Comparison of the functional connectivity (FC) among the MnPO, DMN, FPCN, and SN at baseline, dehydration, and rehydration status with repeated-measures ANOVA (*p*_corrected_ < 0.05, corrected by 3dClustSim in AFNI). Dotted lines indicate that the significant regions were covered by the gyrus in the 3D rendering map

We next explored how different hydration statuses would affect the functional connectivity (FC) pathway. First, we performed group-level independent component analysis (gICA) and dual regression on all subjects’ rs-fMRI datasets. We identified three spontaneous thought-related FC subnetworks (Fig. 3a), including the DMN, FPCN, and SN. The DMN includes the anterior medial prefrontal frontal cortex (amPFC), retrosplenial cortex (RSC), posterior cingulate cortex (PCC), posterior inferior parietal lobule (pIPL), hippocampal formation (HF) and parahippocampal cortex (PHC). The FPCN includes, most prominently, dorsolateral PFC (dlPFC) and anterior IPL (aIPL). The most prominent regions of the SN are the anterior insula (AI) and ACC. Second, we manually placed two seeds in the bilateral MnPO and then constructed the FC map between the bilateral MnPO and the three identified networks (i.e., DMN, FPCN, and SN) (Fig. 3a).

To determine the dynamic changes in the functional interactions between MnPO and high-level cognition systems across different dehydration statuses, we used repeated-measures ANOVA to examine the alterations of FC between MnPO and the three functional subsystems among the baseline (normohydration), dehydration and rehydration statuses. Compared with baseline, we found significantly decreased FC of the MnPO during the dehydrated status with the right precuneus (r-Pc) in the DMN, right superior frontal gyrus (r-SFG) in the FPCN, and anterior middle cingulate cortex (aMCC) in the SN (Fig. 3b, Additional file 1: Fig. S3, and Table 1). During rehydration, these connections then significantly increased and returned to baseline (Additional file 1: Fig. S3 and Table 1). In addition, we investigated whether the FCs of the three spontaneous thought-related functional systems were altered by changes in thirst status (Fig. 3b, Additional file 1: Fig. S3 and Table 1). Repeated-measures ANOVA showed significant changes among the three hydration statuses in FC between DMN and the brain regions of aMCC, right middle frontal gyrus (r-MFG), bilateral superior temporal gyrus (STG), right insular and putamen (r-INS and PUT), left Pc and left IPL (l-IPL), and between SN and the bilateral Pc, PCC, left supramarginal gyrus (l-SMG), INS and inferior frontal gyrus (l-IFG), and between FPCN and regions of r-MFG and r-STG. Overall, compared with baseline and rehydration, we found significantly increased FC in the connections of FPCN–DMN, DMN–SN, and within FPCN, while significantly decreased connections were found in the connections within DMN and SN during dehydration. These findings reveal that the interactions among the MnPO and high-level cognition systems are dynamically regulated in response to continuous thirst-related physiological changes.

**Table 1:** Significant functional connectivity changes revealed by one-way repeated-measures ANOVA at baseline, dehydration, and rehydration

**Brain region**		**ANOVA**			
	**Cluster size**	**x, y, z, MNI coordinates of peak**			**F-test**
**Seed: POA**					
*Right Precuneus (r-Pc)*	86	4	-46	42	11.1
*Right Superior Frontal Gyrus (r-SFG)*	83	16	16	54	8.3
*Middle Cingulate Cortex (MCC)*	75	4	-2	34	12.6
**Seed: DMN **					
*Bilateral Middle Cingulate Cortex (MCC)*	450	4	10	42	12.4
*Right Middle Frontal Gyrus (r-MFG)*	403	32	44	22	16.0
*Right Superior Temporal Gyrus (r-STG)*	277	58	-30	16	12.0
*Left Middle Frontal Gyrus (l-MFG)*	142	-32	46	32	9.1
*Right Insular and Putamen (r-INS and PUT)*	138	30	6	6	10.1
*Left Precuneus (l-Pc)*	136	-4	-64	30	11.6
*Left Superior Temporal Gyrus (l-STG)*	92	-58	-30	14	9.8
*Right Medial Frontal Gyrus (r-MeFG)*	91	4	50	16	7.2
*Right Superior Temporal Pole (r-STP)*	90	52	6	0	11.6
*Left Inferior Parietal Lobule (l-IPL)*	77	-40	-52	50	9.2
**Seed: FPCN**					
*Right Middle Frontal Gyrus (r-MFG)*	167	34	52	12	12.4
*Right Superior Temporal Gyrus (r-STG)*	128	60	-28	16	21.3
**Seed: SN**					
*Bilateral Precuneus and Bilateral Posterior Cingulate Cortex (Pc and PCC)*	1237	0	-52	24	13.0
*Left Supramarginal Gyrus (l-SMG)*	238	-62	-30	32	13.6
*Left Insular / Left Inferior Frontal Gyrus (l-INS and l-IFG)*	89	-30	26	-2	11.2

### Thirst behavior is derived from physiological status through the interactions of the MnPO and high-level cognition systems

**Fig. 4.**
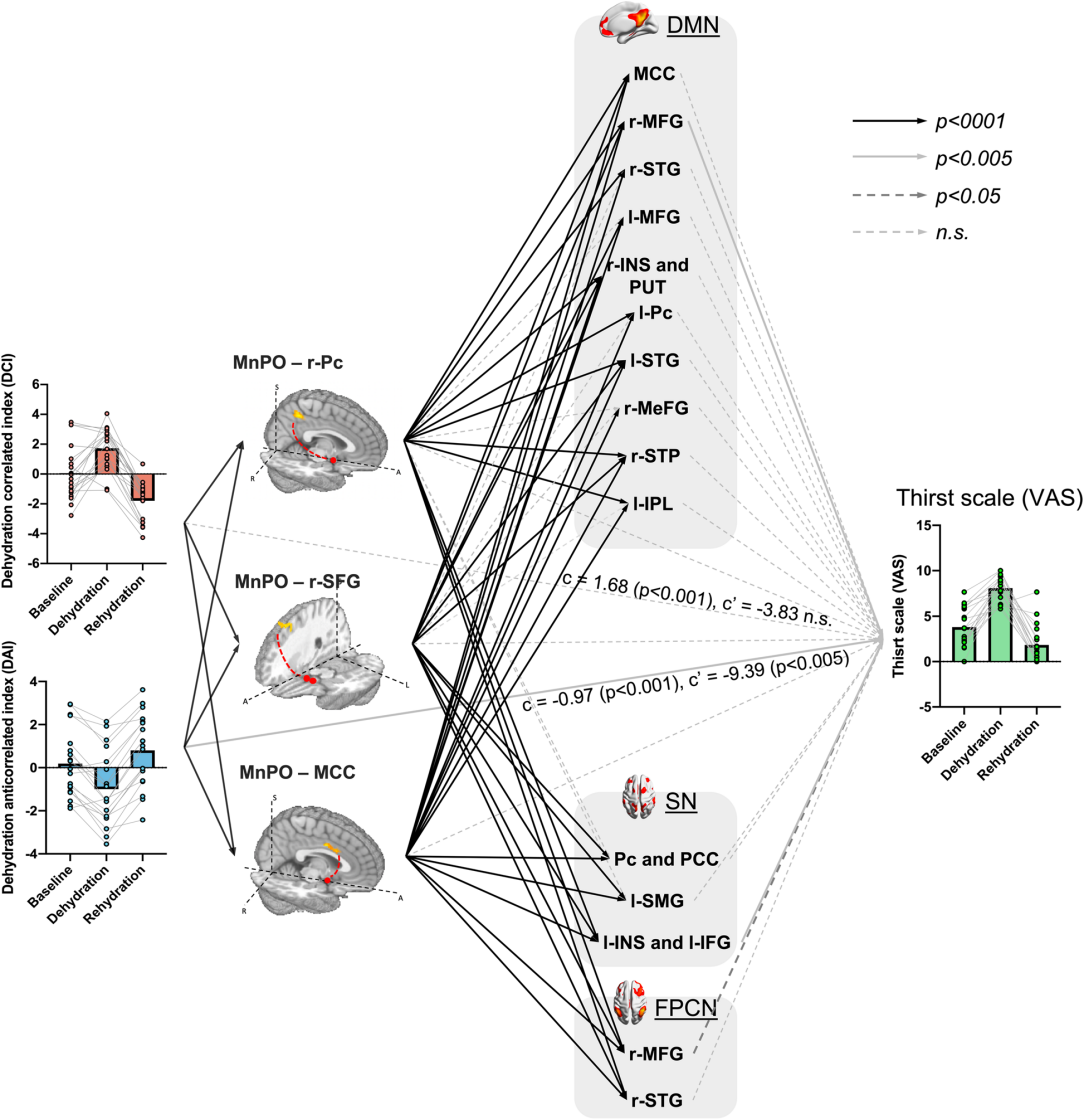
Mediation analysis. The association between hydration-related clinical characteristics (dehydration correlated index, DCI; dehydration anticorrelated index, DAI) and thirst scale (visual analog scale, VAS) was mediated by (1) the connections of MnPO–DMN (r-Pc and PCC), MnPO–FPCN (r-SFG), and MnPO–SN (MCC) and (2) network interactions in multiple mediation analysis.

**Fig. 5.**
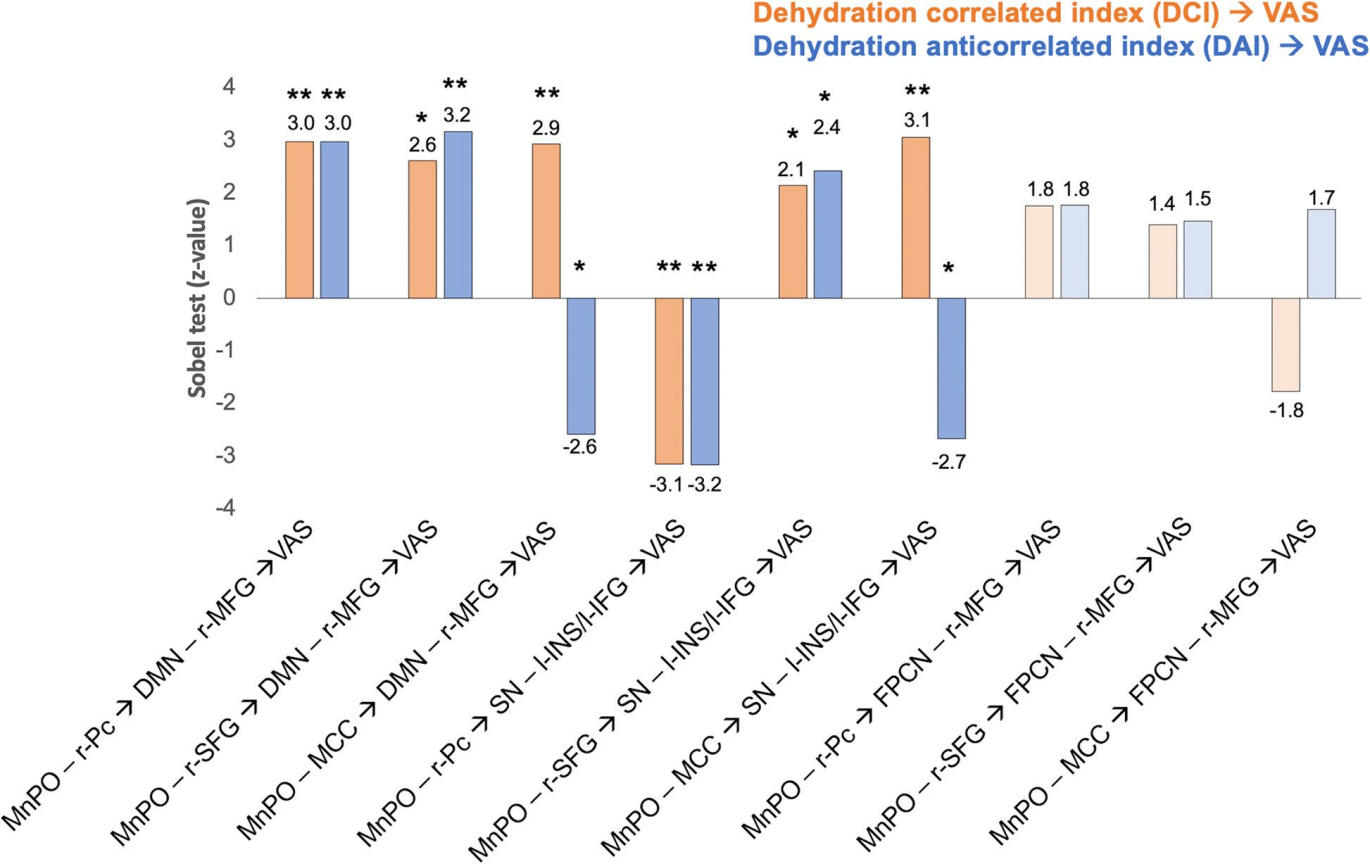
Mediation effect tests (Sobel test) for each mediation path. In each mediation path (dehydration index➔first mediator➔second mediator➔VAS), the significant serial-multiple mediation was estimated by using the Sobel test (z value). (* *p* < 0.05, ** *p* < 0.01, two-tailed hypothesis, FDR corrected)

**Fig. 6.**
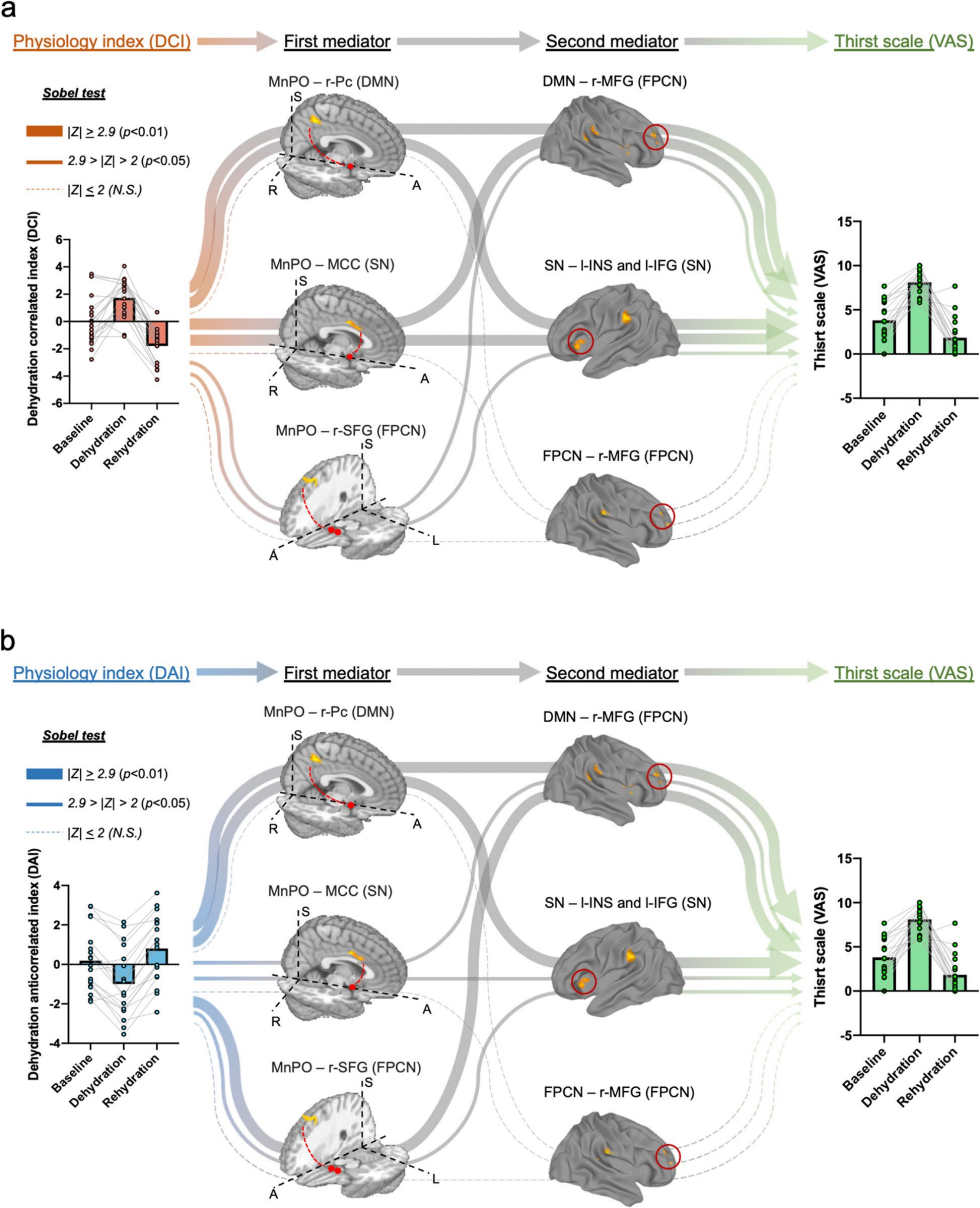
Summary of significant mediation paths from physiological indices. The DCI (**a**), DAI (**b**), and thirst scale (VAS) through MnPO connections and intrinsic network connections (see Additional file 1: Fig. S4 and Additional file 1: Table S1 for the full mediation model). In each mediation path, the solid lines indicate a significant mediation effect, which was estimated by using the Sobel test (*z* value). The *p* value with a two-tailed hypothesis was corrected by FDR correction. The significant paths are shown in thick lines (Sobel test, |*z*| > 2.9, *p* < 0.01) and thin lines (Sobel test, 2.9 > |*z*| > 2, *p* < 0.05), where the dotted lines show no significance

To discover the underlying functional pathways for thirst regulation, we further applied the serial-multiple mediation analysis model [23] to investigate how the physiological components manipulate thirst behavior via the identified brain connections (Fig. 4 and Additional file 1: Fig. S4). Specifically, we first estimated the direct relationships between physiological characteristics and the variations in the thirst scale (visual analog scale, VAS) scores of individual subjects by using a linear mixed-effect regression (LMER) model. We found a significant positive correlation between *DCI* and VAS (*c* = 1.68, *t* = 32.91, *p* < 0.001) (Fig. 5 and Additional file 1: Table S1) and a significant negative correlation between *DAI* and VAS (*c* = −0.97, *t* = −12.09, *p* < 0.001, Fig. 5 and Additional file 1: Table S1). We used a serial multiple mediation model with *DAI* and *DCI* as *independent variables X*, VAS as *dependent variable Y*, and the FCs with significant changes between different thirst statuses as mediator variables *M* to evaluate the potential mediation effect of physiological changes on the thirsty scale via the consecutive pathways from the thalamic thirst center to the intrinsic cortical networks. Considering that the MnPO may serve as an intermediate for projecting the sensory information to the high-order cortices in the multiple mediation model, we set the FCs from the MnPO to the three intrinsic FC networks as the first-level *mediator variables M*_1_ and the FCs among the three networks as the second-level *mediator variables M*_2_ (Fig. 6). Specifically, the relationship between *DAI* and VAS was partially mediated (*c* ^′^ =-9.39, *t* = −3.07, *p*<0.005) through the first (FCs of MnPO–r-Pc, MnPO–r-SFG, and MnPO–MCC) and second mediators (FCs of DMN–r-MFG, SN–l-INS and l-IFG, and FPCN–r-MFG), and the relationship between the *DCI* and VAS was fully mediated (*c* ^′^ = −3.83, *t* = −1.56, n.s.) through the first (FCs of MnPO–r-Pc, MnPO–r-SFG, and MnPO–MCC) and second mediators (FCs of DMN–r-MFG, SN–l-INS and l-IFG, and FPCN–r-MFG) (Fig. 4 and Additional file 1: Table S1).

Additionally, for each mediation path, the Sobel test [23] was adopted to test its significance (Figs. 4 and 6). A strong mediation effect (|*z* value| ≥ 2.9, *p*_corrected_ < 0.01) (Figs. 5 and 6) was found in the path from physiological indices (*DCI* and *DAI*) to the thirst scale (VAS) through the connection of MnP–r-Pc (DMN) as the first mediator and the connections of DMN–r-MFG and SN–l-INS as second mediators. Moreover, while the relationship between *DCI* and VAS was strongly positively mediated through the first mediator of MnPO–MCC (SN) connectivity and the second mediators of SN–l-INS (*z* = 3.1) and DMN–r-MFG (*z* = 2.9) connectivity, the path between DAI and VAS was negatively mediated (*z* = −2.6 and −2.7) through the same mediators (Fig. 6a). The relationship between *DAI* and VAS was strongly (*z* = 3.2) mediated through the first mediator of MnPO–r-SFG (FPCN) and the second mediator of DMN–r-MFG, which was stronger than the same path between *DCI* and VAS (*z* = 2.6, *p* < 0.05) (Fig. 6b). Taken together, our findings demonstrate that thirst behavior is derived from physiological status through the interactions of the MnPO and high-level cognition systems.

## Discussion

What motivates us to drink? Our findings unveiled a mechanistic neural framework to assess subjective thirst from the alterations of physiological status and brain-wide network interactions. Specifically, the subjective experience of thirst is derived from dehydration-related physiological changes (DCI and DAI) through a series of functional pathways connecting the thalamic physiological thirst core with higher-order cortical centers and through further interplay among large-scale brain networks that are involved in spontaneous thought coordination. These findings reveal that the neural circuits centered in both the thalamic and large-scale cortical networks are responsible for generating subjective thirst to maintain fluid balance in humans.

In our study, the dehydration effect was observed as the major component of the two physiological indices (DCI and DAI) and the thirst behavior score (VAS). Since thirst is a subjective sensation, the VAS has been the most commonly used scale to quantify the subjective measurement of thirst. Previous studies have suggested that a common neural mechanism may underlie both physiological status and thirst intention because they are co-occurring and causally interactive [9, 24].

To further identify the underlying neural pathways, we focused on one of the well-identified key regions associated with thirst regulation — the MnPO — and three well-characterized networks related to spontaneous thought. MnPO, as the key to thirst regulation, plays a critical role in thirst processing, which integrates thirst signals from SFO and VOLT and transmits them to downstream brain regions to induce thirst [7]. Functional connectivity of the MnPO with three higher-order cortical regions, including the aMCC, right Pc, and SFG, was found to decrease under dehydration and return to baseline after rehydration. These regions were shown previously to be associated with drinking-related activations [8–11] and fMRI response alterations between thirsty and oversatiated conditions [25], indicating potential involvement in thirst perception. In addition, evidence from resting-state fMRI research suggests that these three regions are among the cores of the major large-scale brain networks with relevance to spontaneous thought [17, 26–28]. Specifically, the Pc plays a key role in DMN and is implicated in internally oriented cognition [26], the aMCC is considered to be a hub of the salience detection network with bilateral anterior insula [27], and the SFG is one of the core regions of the FPCN. Taken together, our findings suggest that the physiological thirst information conveyed by the MnPO may broadcast to large-scale brain networks through its projections with these cortical hubs.

Thirst is a spontaneous thought process that occurs unconsciously with or without minimal external stimulus [22]. Critically, spontaneous thought can be considered a stream of internally oriented thought constrained by either automatic or deliberate resources. Interactions among large-scale brain networks, especially the DMN, SN, and FPCN, have been recognized as the neural underpinnings of the dynamics of spontaneous thought [17]. The DMN was originally identified to be deactivated across a range of goal-oriented tasks and has been associated with intrinsic, stimulus-independent mental processes [29, 30], as well as contributing to internally oriented cognition [31]. The SN is important for the detection and processing of salient inputs [19], including not only the external stimulus that is accessible to conscious awareness but also the integrated signals from visceral, homeostatic, and autonomic stimuli [32, 33], and thus may act as sources of automatic constraints on spontaneous thought. The FPCN, which plays a central role in cognitive control and can couple with either the internally or externally oriented network to implement goal-directed behaviors [17, 34, 35], is hypothesized to exert deliberate constraints on spontaneous thought [36].

In the context of subjective thirst, we found significantly increased connectivity within the FPCN but decreased connectivity within the SN during dehydration, which may indicate their competing roles acting as automatic and deliberate constraints in modulating spontaneous thought. The SN received dehydration-related physiological signals projected from the MnPO, and these automatic signals integrated within the SN were relayed further to other brain networks to constrain spontaneous activities. Therefore, we postulate that decreased internal connectivity in the SN may reflect its passivated activity or a potential protective mechanism to inhibit self-communications to prevent excessive amplification of dehydration-related inputs as it continues to receive physiological signals during prolonged experience of thirst.

On the other hand, thirst is an unpleasant sensation that motivates our drinking behavior to eliminate this negative feeling. As water is not always readily available and drinking is not merely a voluntary reflex action, externally directed thought should consequently involve drinking behavior. In our experiment, during which water was not available for 12 h, the increased connectivity within the FPN may reflect its active involvement in deliberately shifting attention from internally oriented thought to inhibit the participants’ urge to drink water. Moreover, during dehydration, we found weakened anti-correlations between the DMN with both SN and FPCN, along with reduced connectivity within the DMN, suggesting that the SN and FPCN may exert automatic and deliberate control over spontaneous activities through uncoupling their interactions with the DMN, which may lead to suppressed communications within the DMN to inhibit rumination over the unpleasant feeling of thirst. Interestingly, serial-multiple mediation analysis revealed that inputs of physiological signals of fluid balance were first processed via the connectivity of the MnPO with the cortical hubs and then passed further to the three large-scale intrinsic networks, among which the functional interactions were dynamically modulated to generate the subjective experience of thirst.

We recognize some limitations in this study. The reason we chose 1.0L water for rehydration is based on the literature [37]. However, there are individual differences in the amount of water needed for adequate hydration and the lack of normalization to body weight or body composition in the rehydration strategy might be a limitation for the study. Second, MnPO is one of the nuclei in the preoptic area of the hypothalamus which is very small and close to other nuclei. Precise localization of MnPO in anatomic and functional MRI might not always be reliable due to the limitations of image resolution, motion as well as the post-processing software.

**Fig. 7.**
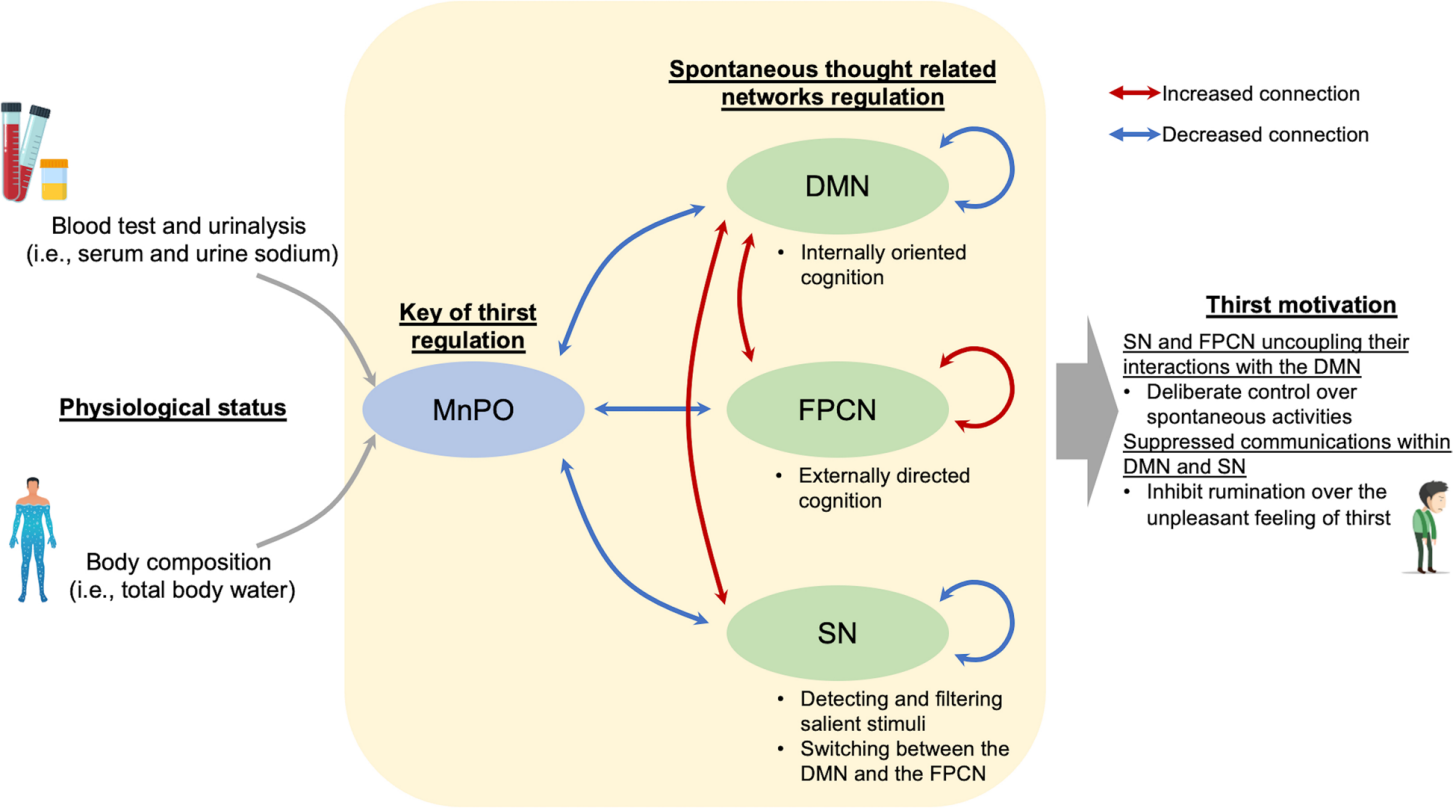
Model of the functional neural pathway for the thirst regulation mechanism. Changes in physiological status inhibit the connection between the MnPO and spontaneous thought-related networks (DMN, FPCN, and SN). This enhances the connections between these networks to generate the thirst motivation

In summary, by using functional connectivity and serial-multiple mediation analysis, we delineated the neural circuits for transforming physiological signals into thirst behavior (Fig. 7). Changes in physiological signals such as serum osmolality detected by the osmoreceptors housed in the CVO inhibit the connection between the MnPO and the brain intrinsic networks (first mediators) and consequently enhance the connections among these networks (second mediators) to generate thirst sensations. Together, our results show that the processing of thirst is not limited within the thalamo-cortical circuit, as previously suggested [14, 38], but rather involves large-scale intrinsic brain networks, especially those implicated in spontaneous thought, which may play critical roles in constraining and modulating thirst-related behaviors. This may provide critical insights into how large-scale intrinsic brain networks can transform sensory inputs from physiological needs into human spontaneous behavioral responses.

## Methods

### Data information

Twenty healthy volunteers (10 women and 10 men) with ages ranging from 24 to 40 (mean 32.1 ± 4.1) years were prospectively enrolled in this study. All subjects were right-handed without a history of any psychiatric, neurologic, or medical illness. Study procedures were approved by the Institutional Review Board of Chang Gung Medical Foundation, and informed written consent was obtained from each participant before enrollment.

To track how the human brain responds to water-related psychological changes, we designed an experiment that simultaneously collected MRI scans and diverse psychological data under different hydration statuses within 13 h in a longitudinal manner. As shown in Fig. 2a, our experiment set three fixed time points in the timeline for data collection, each representing one hydration status. At the first time point, the participants were in a *normohydration status* without any limitations of food or fluid intake in the past several hours. The second time point was set at 12 h after the first one, during which the participants were prohibited from eating or drinking anything with a water content for 12 h to achieve a *dehydration status*. The third time point represents the *rehydration status,* which was set to one hour after the participants were asked to drink 1.0 L water for rehydration.

The MRI data were collected with a 3-T Siemens Verio MRI system (Siemens Medical System, Erlangen, Germany) using a 32-channel head coil. All subjects were instructed to stay awake and relaxed with their eyes closed during the scanning. The MRI protocol was performed in all three hydration statuses, including baseline, dehydration, and rehydration. Rs-fMRI data were acquired with a gradient EPI sequence using the following parameters: TR = 2500 ms, TE = 27 ms, FOV = 220 mm, matrix = 64×64×36, voxel size = 3.4×3.4×4 mm^3^, and total volumes = 240 (10 min 7 sec). 3D MP-RAGE anatomical images were acquired using a gradient echo sequence with the following parameters: TR = 1900 ms, TE = 2.98 ms, FOV = 230 mm, matrix = 220×256, slice number = 160, and voxel size = 0.9×0.9×0.9 mm^3^.

The categorical rating thirsty scale was used to assess the sensation of thirst [39]. Briefly, all subjects were asked to answer the question of “How thirsty do you feel now?” by placing a mark on a 10-cm horizontal line anchored by the phrases “not at all” and “very thirsty” at bilateral extremes. The physiological status of hydration was evaluated with three serum parameters (B) [i.e., glucose, sodium (Na (B)) and osmolality (osmolality (B))] and four urine parameters (U) [i.e., sodium (Na (U)), osmolality (osmolality (U)), creatinine (creatinine (U)) and specific gravity (SP gravity)]. Body water composition, including TBW, ICW, and ECW, was measured by a bioelectrical impedance body composition analyzer (X-SCAN PLUS II, Jawon Medical., Korea).

### Data preprocessing

The rs-fMRI data were preprocessed with AFNI [40]. Allowing for the equilibration of the magnetic field, the first 10 volumes were discarded for each scan of each subject. The remaining rs-fMRI data were preprocessed with the following steps: (1) slice timing; (2) head motion correction; (3) normalization to the MNI atlas with a resolution of 2×2×2 mm^3^; (4) spatial smoothing with a 6-mm full-width half-maximum (FWHM) Gaussian kernel; (5) mean signal removal (including white matter, cerebrospinal fluid (CSF), whole-brain averaged signal, and six head motion parameters) by using the linear regression model; and (6) temporal bandpass filtering (0.01–0.1 Hz). Additionally, to further remove head motion effects, we scrubbed the data and deleted one volume before and after each bad frame to control the global measure of framewise displacement (FD > 0.5 mm) [41, 42]. After scrubbing, subjects with less than 120 volumes were excluded, and one subject was removed from further analysis. Finally, the spike was removed from the blood oxygen level-dependent (BOLD) time series by using 3dDespike in AFNI.

All collected physiological indices of hydration were normalized to z scores and then pooled into the PCA model to extract the factors that primarily contribute to the overall hydration physiological status. In our work, the factors (i.e., eigenvectors) with eigenvalue scores larger than 1 were retained as PCs, and all physiological indices were projected on the PCs to generate the new physiological representation (i.e., PC scores) with low dimensionality. Notably, all of the following experiments on physiological indices were conducted on the PC scores instead of the original physiological data to reduce the analysis complexity.

### Functional connectivity map construction

All rs-fMRI data (total 57 scans: 19 subjects × 3 scans) were used to generate resting-state FC network maps by using the gICA. The number of components was set to 15 based on previous studies [43, 44]. To investigate the individual-level and group-level FC networks simultaneously, dual regression was specifically adopted in our work to identify the subject-specific spatial distribution map of each available component [45], and a one-sample *t* test was then performed on all individual-level maps of each component to identify the corresponding group-level spatial distribution. Based on all detected group-level FC components, three well-known spontaneous-related networks were selected by visual inspection, which included the DMN, FPCN, and SN [17].

As a hub region for thirst regulation, the MnPO was also included as a region of interest (ROI) in our work to explore its FC alterations with three identified thought-related functional subnetworks under three hydration statuses. To this end, two spherical seeds with diameter = 3 mm were first manually placed in the bilateral MnPO (see Fig. 1) in the Montreal Neurological Institute coordinates of [±3.5, 0.6, −13.2] according to [46]. MnPO-based FC maps were then constructed by estimating the Pearson correlation of BOLD time courses between the MnPO seeds and voxels in the DMN, FPCN, and SN.

### Comparisons of psychological state and brain functional connectivity under different hydration statuses

To explore whether psychological states and functional connectivity are altered under different hydration statuses, repeated-measures ANOVA was performed on (1) thirstiness scores, (2) psychological PC scores, (3) FCs within three thought-related functional subnetworks (i.e., DMN, FPCN, and SN), and (4) FCs between the three networks and bilateral MnPO. For thirst scores and physiological data, the significance levels were set to *p* < 0.05 after false discovery rate (FDR) correction. For FCs, the significance levels were set to *p*_corrected_ < 0.05 (uncorrected *p* = 0.01, cluster size = 600 mm^3^) with 3Dclustsim correction in AFNI.

### Uncovering functional pathways for thirst regulation with a multiple mediation analysis model

In addition to the above cross-sectional comparisons of physiological state and FCs between different hydration statuses, we further investigated how the brain integrates diverse regions to respond to physiological changes. To this end, we first calculated the direct relationships between two physiological indices (i.e., *DCI* and *DAI*) and VAS. In our work, the LMER model [47] was used instead of the general linear regression model to estimate the correlation coefficient between two variables for its ability to handle longitudinal data. In each LMER model, age and gender were added as the covariates, and random intercept and subject effects were included as the characterization of the temporal correlation. Second, the serial multiple mediation analysis model was adopted to estimate the indirect relationship between the physiological state and thirsty scale with the MnPO and the three identified thought-related networks as mediations [23]. In this model (see Fig. 5), two physiological PC scores (i.e., *DCI* and *DAI*) were entered as *independent variables X*, and VAS was entered as *dependent variable Y*. The significantly changing FCs between the MnPO and the three intrinsic networks across the difference groups were entered as the first-level mediator variables *M*_1_, and the significantly changing FCs within DMN, FPCN, and SN across the difference groups were entered as the second-level *mediator variables M*_2_. Similarly, the LMER model was used to evaluate the correlation coefficient between the input and output variables. For each pathway (Fig. 5), we utilized the bootstrapping test 1000 times to test the significance. Finally, the mediation effect of each significant path was evaluated using the Sobel test [23]. The *z* value (Sobel test) is estimated:

$$z\ value=\frac{a_1\times {b}_2\times d}{\sqrt{{a_1}^2\times {b_2}^2\times SE(d)+{a_1}^2\times {d}^2\times SE\left({b}_2\right)+{b_2}^2\times {d}^2\times SE\left({a}_1\right)}},$$

where *a*_1_ is the mean coefficient of the path from *X* to *M*_1_, *d* is the mean coefficient of the path from *M*_1_ to *M*_2_, *b*_2_ is the mean coefficient of the path from *M*_2_ to Y, and SE represents the standard error.

## Supplementary Information

**Additional file 1: Figure S1.** Repeated-measures ANOVA of physiological indexes. **Figure S2.** Principal component variances and total variances explained of PCA analysis in physiological indexes. **Figure S3.** Comparison of the whole brain functional connectivity of MnPO and brain regions among different hydration status. **Figure S4.** Path diagram of the multiple-mediation model for this study. **Table S1.** Path analysis.

## Data Availability

All data generated or analyzed during this study are included in this published article and its supplementary information files.

## Abbreviations

ACC: Anterior cingulate cortex
AI: Anterior insular
BOLD: blood oxygen level-dependent
CSF: Cerebrospinal fluid
DAI: Dehydration-Anticorrelated Index
DCI: Dehydration-related Index
DMN: Default mode network
ECW: Extracellular water
FC: Functional connectivity
FD: Framewise displacement
FDR: False discovery rate
FPCN: Frontoparietal control network
FWHM: Full-width half-maximum
gICA: Group-level independent component analysis
HF: Hippocampal formation
ICW: Intracellular water
IFG: Inferior frontal gyrus
INS: Insular
IPL: Inferior parietal lobule
LMER: Linear mixed-effect regression
MCC: Middle cingulate cortex
MFG: Middle frontal gyrus
MnPO: Median preoptic nucleus
OVLT: Oorganum vasculosum lamina terminalis
Pc: Precuneus
PC: Principal component
PCA: Principal component analysis
PCC: Posterior cingulate cortex
PFC: Prefrontal frontal cortex
PHC: Parahippocampal cortex
PUT: Putamen
ROI: Region of interest
rs-fMRI: Resting-state functional magnetic resonance imaging
RSC: Retrosplenial cortex
SFG: Superior frontal gyrus
SFO: Subfornical organ
SMG: Supramarginal gyrus
SN: Salience network
SP gravity: Specific gravity
STG: Superior temporal gyrus
TBW: Total body water
VAS: Visual analog scale
