# Supplementary material for: Human thirst behavior requires transformation of sensory inputs by intrinsic brain networks
Source: BMC Biol. 2022 Nov 10;20:255. doi: 10.1186/s12915-022-01446-5 (PMC9650886; doi:10.1186/s12915-022-01446-5)
Supplement: Supplementary file 1 — Additional file 1: Figure S1. Repeated-measures ANOVA of physiological indexes. Figure S2. Principal component variances and total variances explained of PCA analysis in physiological indexes. Figure S3. Comparison of the whole brain functional connectivity of MnPO and brain regions among different hydration status. Figure S4. Path diagram of the multiple-mediation model for this study. Table S1. Path analysis. [file 12915_2022_1446_MOESM1_ESM.docx]

Additional files 1:


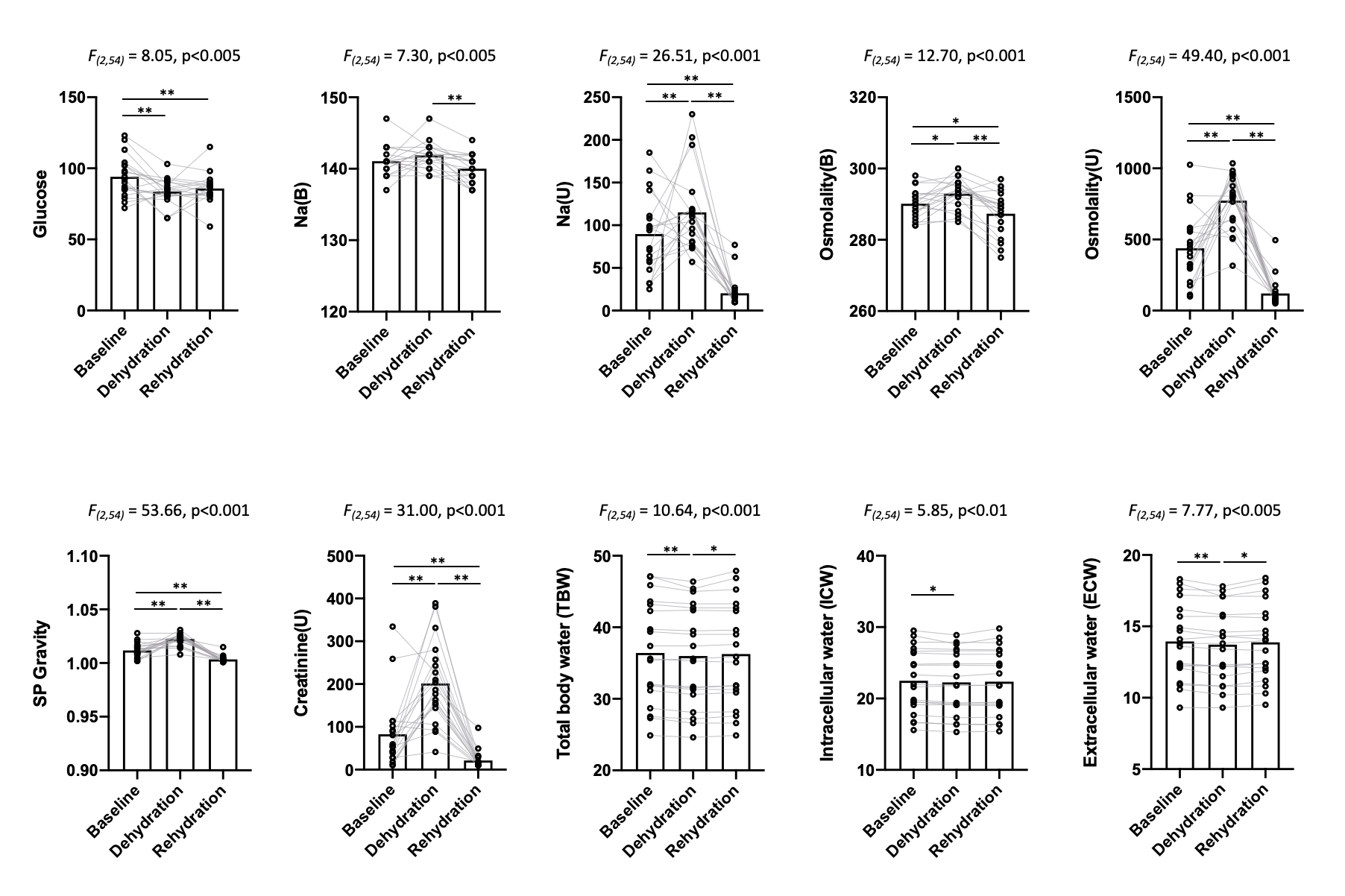


Figure S1. Repeated-measures ANOVA of physiological indexes. The F-value of ANOVA is labeled above each bar graph. (* p<0.05, ** p<0.02, FDR corrected).

Figure S2. Principal component variances (eigenvalue) and total variance explained (%) of PCA analysis in physiological indexes.


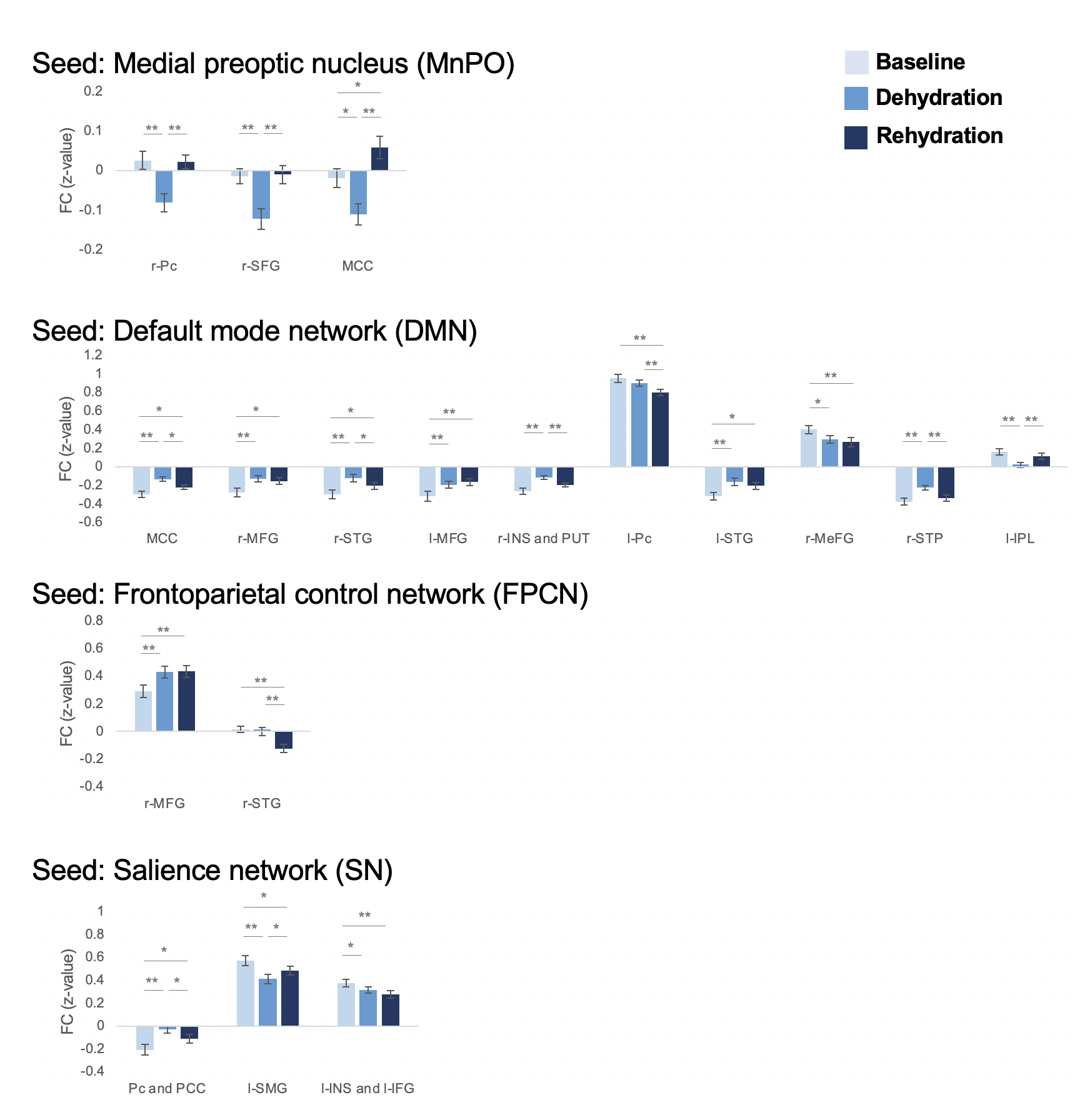


Figure S3. Comparison of the whole brain functional connectivity (FC) of MnPO and brain regions in DMN, FPCN, and SN among baseline, dehydration, and rehydration days with repeated-measures ANOVA analysis.

Figure S4. Path diagram of the multiple-mediation model for this study. X: physiological indexes (DCI and DAI). M_1_: first mediator (MnPO related connections). M_2_: second mediator (spontaneous thought related connections). Y: Thirst scale (VAS)

Table S1. Path analysis (CI = confidence interval)

|  | **Coefficient** | **CI** | **p-value** |  | **Coefficient** | **CI** | **p-value** |
| --- | --- | --- | --- | --- | --- | --- | --- |
| **Directly effect** |  |  |  |  |  |  |  |
| *DCI 🡪 Thirst scale* | 1.683 | 1.583, 1.783 | ***p*<0.001** | *DAI 🡪 Thirst scale* | -0.975 | -1.133, -0.816 | ***p*<0.001** |
| **Mediation effect (1st moderator)** |  |  |  |  |  |  |  |
| *DCI 🡪 MnPO–r-Pc* | 0.030 | 0.028, 0.032 | ***p*<0.001** | *DCI 🡪 MnPO–r-SFG* | 0.005 | 0.003, 0.007 | ***p*<0.001** |
| *DCI 🡪 MnPO–MCC* | -0.046 | -0.052, -0.040 | ***p*<0.001** | *DAI 🡪 MnPO–r-Pc* | 0.129 | 0.126, 0.131 | ***p*<0.001** |
| *DAI 🡪 MnPO–r-SFG* | 0.093 | 0.090, 0.096 | ***p*<0.001** | *DAI 🡪 MnPO–MCC* | 0.025 | 0.016, 0.035 | ***p*<0.001** |
| **Mediation effect (2nd moderator)** |  |  |  |  |  |  |  |
| *DCI 🡪 Thirst scale* | -3.829 | -8.6329, 0.975 | n.s. | *DAI 🡪 Thirst scale* | -9.3892 | -15.383, -3.398 | ***p*<0.005** |
| *r-Pc🡪 Thirst scale* | 25.745 | -12.072, 63.562 | n.s. | *r-SFG🡪 Thirst scale* | -50.888 | -112.961, 11.184 | n.s. |
| *MCC 🡪 Thirst scale* | 16.070 | -16.868, 49.008 | n.s. | *DMN–MCC 🡪 Thirst scale* | 7.401 | -18.192, 32.994 | n.s. |
| *DMN–r-MFG 🡪 Thirst scale* | -35.745 | -57.702, -13.788 | ***p*<0.005** | *DMN–r-STG 🡪 Thirst scale* | -21.294 | -54.025, 11.437 | n.s. |
| *DMN–l-MFG 🡪 Thirst scale* | -13.647 | -54.999, 27.706 | n.s. | *DMN–r-INS and PUT🡪 Thirst scale* | 11.195 | -31.733, 54.123 | n.s. |
| *DMN–l-Pc🡪 Thirst scale* | -4.254 | -21.594, 13.085 | n.s. | *DMN–l-STG 🡪 Thirst scale* | -3.621 | -18.264, 11.021 | n.s. |
| *DMN–r-MeFG 🡪 Thirst scale* | 2.660 | -11.043, 16.364 | n.s. | *DMN–r-STG 🡪 Thirst scale* | 4.121 | -28.804, 37.046 | n.s. |
| *DMN–l-IPL🡪 Thirst scale* | -22.297 | -57.780, 13.185 | n.s. | *SN– Pc 🡪 Thirst scale* | 11.214 | -12.870, 35.298 | n.s. |
| *SN–l-SMG 🡪 Thirst scale* | 24.249 | -35.419, 83.916 | n.s. | *SN–l-INS and l-IFG 🡪 Thirst scale* | 23.267 | 9.000, 37.535 | ***p*<0.005** |
| *FPCN–r-MFG 🡪 Thirst scale* | 22.551 | 0.415, 44.687 | ***p*<0.05** | *FPCN–r-STG 🡪 Thirst scale* | -5.350 | -29.081, 18.381 | n.s. |
| *MnPO–r-Pc🡪 DMN–MCC* | -0.2847 | -0.359, -0.210 | ***p*<0.001** | *MnPO–r-SFG🡪 DMN–MCC* | -0.304 | -0.371, -0.236 | ***p*<0.001** |
| *MnPO–MCC 🡪 DMN–MCC* | -0.208 | -0.264, -0.151 | ***p*<0.001** | *MnPO–r-Pc🡪 DMN–r-MFG* | -0.177 | -0.219, -0.135 | ***p*<0.001** |
| *MnPO–r-SFG🡪 DMN–r-MFG* | -0.508 | -0.544, -0.473 | ***p*<0.001** | *MnPO–MCC 🡪 DMN–r-MFG* | 0.090 | 0.069, 0.111 | ***p*<0.001** |
| *MnPO–r-Pc🡪 DMN–r-STG* | -0.485 | -0.564, -0.406 | ***p*<0.001** | *MnPO–r-SFG🡪 DMN–r-STG* | 0.025 | -0.053, 0.102 | n.s. |
| *MnPO–MCC 🡪 DMN–r-STG* | -0.312 | -0.357, -0.268 | ***p*<0.001** | *MnPO–r-Pc🡪 DMN–l-MFG* | 0.017 | -0.014, 0.048 | n.s. |
| *MnPO–r-SFG🡪 DMN–l-MFG* | -0.635 | -0.664, -0.607 | ***p*<0.001** | *MnPO–MCC 🡪 DMN–l-MFG* | 0.137 | 0.108, 0.166 | ***p*<0.001** |
| *MnPO–r-Pc🡪 DMN–rINS and PUT* | -0.395 | -0.446, -0.343 | ***p*<0.001** | *MnPO–r-SFG🡪 DMN–rINS and PUT* | -0.442 | -0.498, -0.386 | ***p*<0.001** |
| *MnPO–MCC 🡪 DMN–rINS and PUT* | 0.120 | 0.086, 0.155 | ***p*<0.001** | *MnPO–r-Pc🡪 DMN–l-Pc* | -0.282 | -0.321, -0.242 | ***p*<0.001** |
| *MnPO–r-SFG🡪 DMN–lPc* | 0.025 | -0.012, 0.061 | n.s. | *MnPO–MCC 🡪 DMN–lPc* | -0.091 | -0.121, -0.061 | ***p*<0.001** |
| *MnPO–r-Pc🡪 DMN–l-STG* | 0.145 | 0.076, 0.214 | ***p*<0.001** | *MnPO–r-SFG🡪 DMN–l-STG* | -0.464 | -0.527, -0.401 | ***p*<0.001** |
| *MnPO–MCC 🡪 DMN–l-STG* | -0.148 | -0.206, -0.089 | ***p*<0.001** | *MnPO–r-Pc🡪 DMN–r-MeFG* | -0.019 | -0.075, 0.038 | n.s. |
| *MnPO–r-SFG🡪 DMN–r-MeFG* | 0.040 | -0.009, 0.089 | n.s. | *MnPO–MCC 🡪 DMN–r-MeFG* | 0.266 | 0.210, 0.322 | ***p*<0.001** |
| *MnPO–r-Pc🡪 DMN–r-STP* | 0.181 | 0.123, 0.239 | ***p*<0.001** | *MnPO–r-SFG🡪 DMN–r-STP* | -0.682 | -0.736, -0.628 | ***p*<0.001** |
| *MnPO–MCC 🡪 DMN–r-STP* | -0.187 | -0.229, -0.145 | ***p*<0.001** | *MnPO–r-Pc🡪 DMN–l-IPL* | 0.249 | 0.203, 0.294 | ***p*<0.001** |
| *MnPO–r-SFG🡪 DMN–l-IPL* | 0.016 | -0.027, 0.059 | n.s. | *MnPO–MCC 🡪 DMN–l-IPL* | 0.4201 | 0.384, 0.456 | ***p*<0.001** |
| *MnPO–r-Pc🡪 SN–Pc* | -0.016 | -0.082, 0.050 | n.s. | *MnPO–r-SFG🡪 SN–Pc* | -0.143 | -0.211, -0.075 | ***p*<0.001** |
| *MnPO–MCC 🡪 SN–Pc* | -0.353 | -0.402, -0.305 | ***p*<0.001** | *MnPO–r-Pc🡪 SN–l-SMG* | -0.026 | -0.070, 0.018 | n.s. |
| *MnPO–r-SFG🡪 SN–l-SMG* | -0.320 | -0.359, -0.281 | ***p*<0.001** | *MnPO–MCC 🡪 SN–l-SMG* | 0.068 | 0.038, 0.099 | ***p*<0.001** |
| *MnPO–r-Pc🡪 SN–l-INS and l-IFG* | -0.670 | -0.735, -0.604 | ***p*<0.001** | *MnPO–r-SFG🡪 SN–l-INS and l-IFG* | 0.115 | 0.054, 0.177 | ***p*<0.001** |
| *MnPO–MCC 🡪 SN–l-INS and l-IFG* | -0.316 | -0.360, -0.272 | ***p*<0.001** | *MnPO–r-Pc🡪 FPCN-r-MFG* | 0.300 | 0.251, 0.348 | ***p*<0.001** |
| *MnPO–r-SFG🡪 FPCN-r-MFG* | 0.065 | 0.015, 0.114 | ***p*<0.001** | *MnPO–MCC 🡪 FPCN-r-MFG* | 0.467 | 0.438, 0.497 | ***p*<0.001** |
| *MnPO–r-Pc🡪 FPCN-r-STG* | -0.250 | -0.275, -0.226 | ***p*<0.001** | *MnPO–r-SFG🡪 FPCN-r-STG* | 0.165 | 0.142, 0.188 | ***p*<0.001** |
| *MnPO–MCC 🡪 FPCN-r-STG* | 0.042 | 0.030, 0.055 | ***p*<0.001** |  |  |  |  |
